# Detection and colocalization of STEC genetic markers in wheat flour using whole cell digital droplet PCR

**DOI:** 10.3389/fmicb.2026.1854595

**Published:** 2026-07-24

**Authors:** Sophie Butot, Caroline Gaille, Lise Michot, Mathieu Seppey, Caroline Barretto, Solenn Pruvost, Sophie Zuber

**Affiliations:** Institute of Food Safety and Analytical Sciences, Nestlé Research, Lausanne, Switzerland

**Keywords:** colocalization, digital droplet PCR, Shiga toxin-producing *Escherichia coli*, STEC detection, wheat flour, whole cell ddPCR

## Abstract

**Introduction:**

Shiga toxin-producing *Escherichia coli* (STEC) poses a significant food safety risk in wheat-based products. The detection of these pathogens in complex food matrices is challenging due to the labor-intensive nature of conventional methodologies and the variability in definitions across different countries. This study investigated whole cell digital droplet PCR (ddPCR) to detect the co-occurrence of critical STEC genetic markers in inoculated and naturally contaminated wheat flour.

**Methods:**

We optimized the ddPCR protocol for the detection and colocalization of critical STEC genetic markers, namely *stx1*, *stx2*, *eae*, and the Top 7 serogroups. Furthermore, we developed a novel quantitative PCR (qPCR) assay to serve as a generic marker for *E. coli*, specifically targeting the *yecE* gene, for which we demonstrated inclusivity for *E. coli* and exclusivity against non-target species, both *in silico* and *in vivo*. This assay was used in the ddPCR colocalization tests performed throughout the study.

**Results:**

Both qPCR and ddPCR analytical workflows were shown to have a similar LOD50 around 0.9 CFU/g in wheat flour under the tested conditions. Our colocalization analysis was successful in wheat flour inoculated with single strains or a pool of two strains with different genetic make-ups at different inoculation levels. Furthermore, we were able to show detection and genetic association patterns by ddPCR across naturally contaminated retail flour samples.

**Discussion:**

This flexible whole cell ddPCR approach addresses significant limitations of existing STEC detection methods and provides a useful data set to support the development of a more sophisticated molecular analytical method which can be applied to wheat flour to detect and characterize STEC strains.

## Introduction

Shiga toxin-producing *Escherichia coli* (STEC) causes illnesses ranging from mild diarrhea to potentially life-threatening post-diarrheal hemolytic uremic syndrome (HUS), especially in young children. Various STEC serogroups such as O157 are commonly associated with severe illness and HUS, but the primary virulence factors of STEC are two types of Shiga toxins, Stx1 and Stx2, encoded by the *stx1* and *stx2* genes, classified into several subtypes which influence the severity and complications of human illnesses (Tarr et al., 2005; Karmali et al., 1983; Mody et al., 2012; Vishram et al., 2021; Gould et al., 2009; Koutsoumanis et al., 2020; Capps et al., 2021; Melton-Celsa, 2014). STEC has been linked to several outbreaks involving the consumption of products containing raw flour, such as ready-to-bake cookie dough in the USA in 2009 (Neil et al., 2012), dough mix in the US in 2016 (Gieraltowski, 2017; Crowe et al., 2017), retail flour in Canada in 2016–2017 (Morton et al., 2017) and more recently frozen pizzas in France in 2022 (Krug et al., 2026). In the Canadian flour outbreak, STEC O121 was isolated from an open flour sample and from a patient’s home and both isolates grouped together, with only 0–6 whole genome multilocus sequence typing allele differences (Morton et al., 2017). It is important to note that Most Probable Number (MPN) estimates of the concentration of STEC O121 in the recalled Canadian flour were very low with levels estimated at 0.0015 to 0.0043 MPN/g (Gill et al., 2019). In the French frozen pizza outbreak, isolates from pizza dough and flour samples were indistinguishable from clinical outbreak strains by whole genome sequencing (WGS; Krug et al., 2026). Beside outbreak related STEC detection and isolation in flour-based products, the prevalence by polymerase chain reaction (PCR) of STEC in flour was reported to be 10.8% (Boss and Hummerjohann, 2019), 12.0% (Söderlund et al., 2023), 12.9% (Kindle et al., 2019), and 39.0% (Mäde et al., 2017), demonstrating the concern around STEC in flour and flour-based products.

A considerable challenge in STEC detection is the absence of a uniform definition of STEC across different regions. In the USA, in addition to the Top 5 serogroups (O157, O26, O103, O111, O145) screened for in the ISO/TS-13136:2012 (ISO, 2012), the O45 and O121 serogroups are considered adulterants in raw beef, which are referred to as Top 7 serogroups (USDA, 2020). In France, samples are considered presumptive STEC positive when *stx1* and/or *stx2*, *eae* and at least one of the following serogroups are detected: O157, O26, O103, O111, O145 (Top 5), and O80 (DGAL, 2024). In Switzerland, the detection of *stx2* indicates a potentially increased danger of infection (BLV, 2020). In Germany, all STEC are considered as potentially pathogenic, and no distinction is made based on the different *stx* variants or serogroups (BFR, 2020).

Independent of the definition, detecting STEC in food matrices is challenging due to the cumbersome methodology. The current standard method ISO/TS-13136:2012 involves a non-selective enrichment in Buffered Peptone Water (BPW) and describes the screening and the confirmation procedures (ISO, 2012). The screening part consists of the identification of STEC by means of the detection of the major virulence genes of STEC, *stx* and *eae*, and focuses on the Top 5 serogroups in the enriched sample. The confirmation involves plating the presumptive positive enrichment on selective agar, followed by the analysis of single colonies through quantitative PCR (qPCR) to verify the presence of *stx1*, *stx2*, *eae*, and/or one of the Top 5 serogroups. This step is crucial to confirm that the positive qPCR signals detected in the enrichment originate from genes present in the same viable bacterial cell. The confirmation step for detecting STEC is labor-intensive, as individual colonies must be screened by qPCR for the presence of target genes. According to ISO/TS-13136:2012, up to 50 colonies per sample are recommended to be tested. However, this arbitrary selection process poses a significant risk of false-negative results, as STEC colonies may be missed. Mäde and coauthors confirmed 73.3% (11 confirmed isolates out of 15 presumptive positive samples) of flour samples detected positive by qPCR as part of an official food surveillance program in Germany (Mäde et al., 2017). This high confirmation rate was only achieved by a massive screening procedure. This so-called “500 colony procedure” is not a routine analysis and highlights that the current method for detecting and isolating STEC strains is overly complicated and problematic for the food industry.

In this context, the flour milling industry urgently needs more sophisticated molecular STEC analytical methods which can be applied to wheat flour to detect and characterize STEC strains without necessarily going through the confirmatory step. This would allow to quickly channel presumptive positive batches towards baked products such as bread instead of taking the risk of putting unprocessed raw flour on the market. Droplet digital PCR (ddPCR) is becoming the approach of choice for assays looking to detect low copy targets for example in environmental samples (Kokkoris et al., 2021) and ddPCR has previously been employed to detect STEC in DNA extracted from bovine feces (Verhaegen et al., 2016; Paquette et al., 2018) and beef slices (Mancusi et al., 2022). More importantly, intact whole cell ddPCR was investigated to detect the co-occurrence of the *stx* and *eae* genes in a single bacterial STEC cell from inoculated culture broth, milk, apple juice, spinach and ground beef samples (McMahon et al., 2017; He et al., 2020; Capobianco et al., 2020). In ground beef samples, Capobianco and coauthors used Bio-Rad dd-Check STEC detection kit together with the QxIDE Manager software for the acquisition and analysis of whole cell ddPCR data which applies advanced multivariate Poisson distribution statistics to resolve overlapping droplet populations and evaluate molecular colocalization or linkage (Capobianco et al., 2020).

These promising colocalization results in food matrices show that the colocalization concept of whole cell ddPCR may be applied to a wider range of genetic targets in STEC, which present an opportunity to develop a flexible assay that can be used as a risk prioritization tool. As such, whole cell ddPCR has the potential to enhance the differentiation of the genetic background of STEC strains present in a sample to provide preliminary results significantly faster compared to the confirmation by plating described in the ISO/TS-13136:2012 standard. Furthermore, as the ISO/TS-13136:2012 method is under revision and the current genetic STEC targets (*stx*, *eae*, and the Top 5 serogroups) might change in the future (ISO, 2026), it is important to identify genomic regions that are specific to *E. coli* and designing primers that can be used as generic *E. coli* markers in PCR assays. This could allow the development of additional assays targeting other enteropathogenic *E. coli* strains potentially present in flour which are also associated with severe disease, but lacking *stx* genes (Söderlund et al., 2023). The objective of the present work was to develop and optimize a whole cell ddPCR method for the detection and colocalization of critical STEC genetic markers, specifically *stx1*, *stx2*, *eae*, and the Top 7 serogroups, alongside a generic and specific *E. coli* marker, *yecE*, in wheat flour.

## Materials and methods

### Bacterial strain preparation

The bacterial strains (Table 1 and Supplementary Table S1) were stored on cryobeads (Technical Service Consultants Ltd. Lancashire, UK) at −80 °C. A cryobead was transferred to 4 mL of sterile Brain Heart Infusion (BHI; Thermo Fisher Scientific, Oxoid, Waltham, Massachusetts, USA) broth followed by incubation at 37 °C for 24 ± 2 h. A subculture was prepared by inoculating 50 μL of the bead culture in a new tube of 4 mL of BHI broth incubated at 37 °C for 24 ± 2 h, which was then used for sample inoculation or ddPCR. Bacterial subcultures were enumerated on Tryptone Soya Agar (TSA; Thermo Fisher Scientific, Oxoid) and incubated at 37 °C for 24 ± 2 h.

**Table 1:** List of strains used for ddPCR development.

Strain number	Isolate number	Species	Serotype	Virulence genes*	Reference culture collection number
P037	PIR04589	*Escherichia coli*	O26:H11	*stx1, stx2, eae*	Not applicable
P042	PIR05756	*Escherichia coli*	O91:H10	*stx2*	Not applicable
P139	PIR05774	*Escherichia coli*	O157:H7	*eae*	ATCC 700728
P190	PIR05783	*Escherichia coli*	O111:H21	–	ATCC 29552
P201	PIR05791	*Escherichia coli*	O145:–	*stx1, stx2, eae*	CDC 99-3311
P202	PIR05792	*Escherichia coli*	O45:H2	*stx1, eae*	CDC 00-3039
P203	PIR05793	*Escherichia coli*	O121:H19	*stx2, eae*	CDC 02-3211
P204	PIR05794	*Escherichia coli*	O26:H11	*stx1, stx2, eae*	CDC 03-3014
P205	PIR05795	*Escherichia coli*	O103:H11	*stx1, eae*	CDC 06-3008
P206	PIR05796	*Escherichia coli*	O111:H8	*stx1, stx2, eae*	CDC 2010C-3114
P207	PIR05797	*Escherichia coli*	O157:H7	*stx1, stx2, eae*	ATCC 35150

* *yecE* gene is present in all strains listed.

### Whole genome sequencing

The bacterial strains used to identify a generic *E. coli* genomic region and the STEC colonies isolated from flour samples were analyzed by WGS to ensure accurate identification. For this, 1 mL of the bacterial subculture was centrifugated 5 min at 10,000 g and the pellet was further processed for DNA extraction using the Gram-positive and Gram-negative bacteria protocol of the Maxwell RSC system from Promega and the Maxwell RSC cultured Cell kit (Promega, Madison, Wisconsin, USA).

The library preparation for WGS with Illumina short read sequencing was performed on samples starting with 100 ng of genomic DNA input and amplified with 5 PCR cycles, following the user guide without any modifications (Illumina DNA Prep Reference Guide Part # 1000000025416 v09 June 2020) using IDT for Illumina DNA/RNA UD Indexes Set. The quantification for the equimolar pooling was based on the Qubit Hs dsDNA Kit (Thermo Fisher Scientific) and the quality assessed on the TapeStation D1000 ScreenTape (4,200 TapeStation System, Agilent, Santa Clara, California, USA). The equimolar pool was assembled using a Hamilton robotic platform. The sequencing was performed on a NextSeq2000 platform (Illumina, San Diego, California, USA) using P1 600 cycles chemistry, for PE300 run. The pool was spiked with 10% PhiX, loaded at 650 pM. Primary data quality control was performed during the sequencing run to ensure the optimal flow cell loading (cluster density) and check the quality metrics of the sequencing run (QC30) on SAV. The demultiplexing was performed on the NextSeq Instrument by DRAGEN (Version: 3.10.11) using the GenerateFASTQ Workflow to generate FASTQ files for each strain. The bioinformatics workflow involved the characterization of genome sequences at different levels. For each isolate, genome sequence was assembled with SKESA (Souvorov et al., 2018) and evaluated with BUSCO v5 (with auto-lineage; Manni et al., 2021). The prediction of the Sequence Type (ST) based on the seven genes Multi Locus Sequence Typing (MLST) was performed using mlst (GitHub - tseemann/mlst:id: Scan contig files against PubMLST typing schemes) with the PubMLST database.^1^ Additionally, core genome (cg) and whole genome (wg) MLST were employed to group genomes based on their overall similarity using chewBBACA (Silva et al., 2018). Single Nucleotide Polymorphisms (SNPs) were identified through SNP detection using the FDA CFSAN snp pipeline (Davis et al., 2015), which allowed for the comparison of each position in the genome and the precise evaluation of the level of similarity or differences between genomes belonging to a certain cg/wgMLST group. Serotyping determination was performed through homology comparison of sequences in the query genome to a set of serotype-specific O and H antigen genes using STEC Finder (Zhang et al., 2022) and EC-Typer (Bessonov et al., 2021). The in-silico detection of *stx1*, *stx2*, and *eae* genes was accomplished through homology comparisons to the database of virulence factors (Chen et al., 2005). Shiga toxin variants were determined with STEC Finder. This Whole Genome Shotgun project has been deposited at DDBJ/ENA/GenBank under the accession PRJNA1475373.

### Identification of a generic *E. coli* genomic region

A total of 63 *E. coli* and 5 *Shigella* genomes from various sources (Supplementary Table S1), were whole genome sequenced as described above. *Shigella* has been historically considered a distinct albeit closely related genus to *Escherichia*. However, this taxonomic status is being challenged, and *Shigella* sp. could be viewed as a subspecies of *E. coli* (Dif et al., 2025). In the context of this work, however, it is not considered to be part of *E. coli* and is thus classified as a non-targeted organism when seeking a specific genomic region for PCR primer design. Therefore, the 63 genomes belonging to *E. coli* were placed in the positive group while the negative group was made up of the 5 genomes belonging to *Shigella* sequenced in the frame of this project along with 137 public genomes representing various taxa including many known food and non-food pathogens, as well as some non-pathogens, all manually selected from NCBI GenBank (on 16/05/2024), namely: 40 additional *Shigella*, 35 *Salmonella*, 15 *Klebsiella*, 9 *Brochothrix*, 6 *Listeria*, 4 *Streptomyces*, 4 *Pseudoclavibacter*, 4 *Corynebacterium*, 4 *Bacillus*, 3 *Bacteroides*, 2 *Staphylococcus*, 2 *Flavobacterium*, 1 *Riemerella*, 1 *Rhodococcus*, 1 *Nocardiopsis*, 1 *Mycobacteroides*, 1 *Mycobacterium*, 1 *Kocuria*, 1 *Glycomyces*, 1 *Clavibacter*, 1 *Arthrobacter* (GCA accession identifiers are listed in Supplementary Table S2).

A custom script was used to segment these genomes into sequences of 200 base pairs (bp), applying an overlap of 50 bp as the sequence progressed (Seppey, 2026). This approach ensured that each nucleotide in the genome was represented in four shifted sequences. These segments served as the query sequences, representing small regions that could potentially be used for primer design. The query sequences were then searched against each genome in both the positive and negative sets using blastn v2.15 (Altschul et al., 1990), maintaining the default parameters. All blast results were then analyzed to identify and categorize the 200 bp regions that exhibited strong matches in all genomes of the positive set, while showing minimal presence in the negative set. To ensure the most stringent results, the percent identity threshold for matches in the positive set was set at 95%, while any value was accepted for the negative set.

### *YecE* primers and probe design

The forward primer (5′-CATTTCGCATCAGGCAGCA-3′) and the reverse primer (5′-AGTATTGCCCAATGCGCG-3′) were designed with the tool Primer3plus (available at https://www.primer3plus.com) and the TaqMan probe (VIC- or 6-FAM-ACATTCGATGATTTAGT-MGBNFQ) with the Primer Express 3.0 software (Thermo Fisher Scientific, Applied Biosystems) using the generic *E. coli* 200 bp *yecE* sequence identified in this study.

### *In silico* validation of the designed *yecE* primers

The primersearch software from the EMBOSS package 6.5.7 (Rice et al., 2000) using the forward and reverse primer sequences, allowing for 10% mismatches was used to calculate the inclusivity (nb_pos_amplified/tot_nb_pos) and exclusivity (nb_neg_not_amplified/tot_nb_neg) of the primers.

The analysis was performed on genomes of 35,956 *E. coli* available from NCBI RefSeq (accession prefix GCF) at the time of the experiment, serving as the positive set for primersearch validations. Additionally, the genomes of 2,328 *Shigella* and 602 *Escherichia* species that do not belong to *E. coli* from NCBI RefSeq were used as the negative set for the primersearch validations.

### *YecE* qPCR

Inclusivity and exclusivity evaluation is essential for validating microbiological methods used in food testing (ISO, 2016). The designed primers and probe were evaluated by qPCR with a selection of 67 *E. coli* strains to assess inclusivity and 37 non-*E. coli* strains to assess exclusivity (Supplementary Tables S4, S5). The DNA of each bacterial strain was extracted using the protocol described in the WGS section above. All PCR amplifications were performed in a final volume of 25 μL of reaction mixture containing 12.5 μL of TaqMan Universal PCR Master Mix (Thermo Fisher Scientific, Applied Biosystems), 1 μM of each primer, 200 nM of 6-FAM probe, 2.5 μL of TaqMan Exogenous Internal Positive Control (IPC) mix (Thermo Fisher Scientific, Applied Biosystems), 0.25 μL of TaqMan Exogenous Internal Positive Control DNA previously diluted 50 times (Thermo Fisher Scientific, Applied Biosystems) and 5 μL of DNA template. All reactions were performed on an Applied Biosystems 7,500 Fast real-time PCR system. The amplification conditions were the following: 50 °C for 2 min, 95 °C for 10 min, and 40 consecutive cycles of 15 s at 95 °C and 1 min at 60 °C.

### Samples

Retail wheat flours were used in this study. The samples used for the inoculation experiments were previously screened by qPCR using a method based on ISO/TS-13136:2012 (ISO, 2012) as described below to ensure that they were not naturally contaminated with STEC.

### Sample enrichment

Using a method based on ISO/TS-13136:2012, the samples were enriched before being screened for the presence of STEC by qPCR and ddPCR. Briefly, 25 g of wheat flour was distributed on the surface of 225 mL of BPW (Merck, Darmstadt, Germany), prewarmed at room temperature and previously added to a filter stomacher bag (ISO, 2017). After 30 to 60 min standing at room temperature, the diluted sample was homogenized by stomaching for 1 min and incubated 6 h at 37 °C followed by 18 h at 41.5 °C. When not analyzed the same day, aliquots of 2 mL of the enrichment broth were stored at −80 °C until qPCR and ddPCR analysis.

### STEC analysis by qPCR and colony confirmation (based on ISO/TS-13136:2012)

Fifty μL of the enrichment broth from wheat flour was centrifuged at 20,000 g for 2 min. The pellet was resuspended in 200 μL InstaGene Matrix (Bio-Rad, Hercules, California, USA) followed by DNA extraction following supplier’s instructions. The DNA was transferred to a Zymo DNA purification column from the Zymo OneStep PCR Inhibitor Removal Kit (Zymo Research, Irvine, California, USA) to remove potential PCR inhibitors from the extracted DNA, following supplier’s instructions. The DNA extracted from the enrichment broth was analyzed by qPCR to detect the primary virulence genes *stx1*, *stx2*, and *eae*, as well as the Top 7 serogroups (O157, O26, O103, O111, O145, O45 and O121) and the generic *E. coli* marker, *yecE*. The primers and 6-FAM-labelled TaqMan probes used in this study are detailed in ISO/TS-13136:2012 (ISO, 2012), with the exception of those targeting O145, O121, and O45, which are described by USDA (USDA, 2019). All qPCR amplifications were conducted following the previously described conditions for the *yecE* qPCR, with the exception of the O103 qPCR performed at an annealing/extension temperature of 55 °C (Kagkli et al., 2011; EU-RL, 2023).

For samples which were subjected to colony confirmation, serial 10-fold dilutions of the enrichment in tryptone salt solution (TS) containing 1.0 g/L of Tryptone and 8.5 g/L NaCl, were plated on Tryptone Bile X-Glucuronide (TBX; Thermo Fisher Scientific, Oxoid) followed by incubation for 21 ± 3 h at 37 °C. Up to 45 typical and atypical colonies per sample were selected, isolated on TSA and incubated 21 ± 3 h at 37 °C. The DNA from each individual colony was extracted by transferring bacterial material into 200 μL of nuclease free water (Thermo Fisher Scientific, Invitrogen) followed by a heat treatment for 10 min at 97 ± 2 °C at 300 rpm and a centrifugation at 8,000 g for 5 min. The supernatant containing the DNA was then screened for the *stx1*, *stx2* and *eae* genes and the *stx1* or/and *stx2* positive colonies were further screened for the Top 7 serogroups and *yecE* by qPCR, as previously described. To further characterize the *stx* positive colonies, each colony was analyzed by WGS as described above.

### STEC genetic target colocalization by whole cell ddPCR

The flour enrichment sample preparation for ddPCR was based on the protocol Bio-Rad developed for the dd-Check STEC kit. Briefly, 2 mL of enrichment broth was filtered through a 10 μm syringe filter (Pall corporation, Port Washington, New York, USA). One mL of filtered enrichment broth was centrifuged at 6,000 g for 5 min and the supernatant was discarded. The pellet was resuspended in 1 mL of TS.

To assess the colocalization of two STEC targets of interest, duplex ddPCR was performed using primers and TaqMan probes described in the preceding section on STEC analysis by qPCR. This approach involved combining a 6-FAM-labelled probe for one target with a VIC-labelled probe for the second target. To run the ddPCR, 18 μL of ddPCR master mix, including 6 μL of ddPCR Multiplex Supermix (Bio-Rad), 900 nM of each primer and 250 nM of TaqMan probes (Thermo Fisher Scientific) were mixed carefully with 6 μL of the sample (filtered enrichment) in a 96-well plate (Bio-Rad), avoiding bubbles formation. Subsequently, 20 μL were transferred to the middle row of a DG8 cartridge (Bio-Rad) and 70 μL of droplet generation oil for probes (Bio-Rad) were distributed in the dedicated row. A DG8 gasket (Bio-Rad) was placed over the cartridge which was inserted into the QX200 Droplet Generator (Bio-Rad) to generate the droplets by emulsifying the ddPCR mix with the oil. Forty μL of the droplet sample was transferred to a 96-well plate, while ensuring no air was introduced into the mix (slow pipetting for aspiration and expulsion). The plate was heat-sealed using the PX1 PCR plate sealer (Bio-Rad) and subsequently placed in the PTC Tempo Deepwell Thermal Cycler (Bio-Rad) for PCR amplification using the dd-Check STEC Amplification program developed by Bio-Rad. Once the PCR process was completed, the plate was transferred to the QX200 droplet reader (Bio-Rad) and the reading was initiated using the QxIDE Manager software which was developed for the Bio-Rad dd-Check STEC detection kit to determine whether two genetic signals detected within the same droplet belong to the same bacterial cell. The functionalities of this software, the controls and statistical tests performed to determine colocalization or linkage of genetic targets were thoroughly described by Capobianco and coauthors (Capobianco et al., 2020) and AOAC validated in 2024. The software uses advanced multivariate Poisson distribution statistics to resolve overlapping droplet populations and evaluate molecular colocalization. A minimum threshold of 8,000 accepted droplets is necessary for colocalization analysis and the software asks for dilution of the sample if needed.

We included TS negative controls in the PCR plate for each mastermix preparation to ensure the accurate determination of the amplitude of negative droplets. In addition, DNA positive controls were used in preliminary experiments to compare the level of detection of each target between DNA and intact bacterial cells.

### Wheat flour sample inoculation

One mL of P205 (O103, *stx1*, *eae*; Table 1) subculture was added in small droplets across different points to 100 g of wheat flour in a Whirl-Pak Write On Sterilized bag (Filtration Group, Whirl-Pak, Pleasant Prairie, Wisconsin, USA), then mixed thoroughly by a vigorous manual external massage for at least 5 min to obtain a clump free and homogenous mix (Rachon et al., 2016). The Whirl-Pak bag containing this highly contaminated flour was stored open under a laminar flow for 24 h to stabilize the water activity. To prepare flour samples inoculated at different levels, 20 g of this flour highly contaminated with P205 was added to 180 g of non-inoculated flour in a 2 L Nalgene bottle (Nalgene, Rochester, New York, USA) followed by a 1 h homogenization in a Turbula device (Willy A. Bachofen AG, Muttenz, Switzerland). In total, 6 serial dilutions were prepared following the same procedure. From each level of contamination, 5 subsamples of 25 g 10-fold diluted and homogenized in BPW were enumerated on TBX (incubated 24 ± 2 h at 37 °C) and enriched for STEC detection by qPCR and ddPCR as described above. The limit of detection (LOD) of the qPCR and ddPCR targeting *stx1* was estimated using the PODLOD program (version12, PODLOD-ver12.xlsm; ISO, 2016) considering the positive samples per level of inoculation.

To assess the effectiveness of the ddPCR method in discriminating STEC targets from two distinct bacterial strains, additional flour samples were inoculated with 1,000-fold diluted subcultures of either the P190 strain (O111, *yecE*) or the P202 strain (O45, *stx1*, *eae*, *yecE*) as described above and enumerated in triplicates on TSA (P190 flour stock: 3.15 Log_10_ CFU/g and P202 flour stock 2.84 Log_10_ CFU/g). Due to the time-consuming nature of homogenization with the previously described Turbula device, this inoculation method was modified to utilize a blender. The inoculated flour stocks were 2 times 10-fold serially diluted in non-inoculated flour. Subsequently, they were homogenized in a blender at low speed for 5 times 1 min, with a 1-min pause between each mixing to prevent overheating. The flour samples were individually analyzed by qPCR and ddPCR. Additionally, prior enrichment and for each level of inoculation, the flour inoculated with each strain (P190 or P202) were mixed at a ratio 1:1. For each inoculation experiment, a negative control consisting of non-inoculated flour sample was included.

## Results

### Whole genome sequencing

Whole Genome Sequencing performed on the NextSeq platform generated a total yield of 82 Gb with 4,974 Cluster density (K/mm^2^). The overall run quality was high, with approximately 90% of bases achieving a Q30 score and a PhiX spike-in of 12%. All samples were well balanced, with an average of 312 million pass-filter reads per sample, resulting in a mean coverage of the genome of 190X. Assembly quality metrics of the sequenced isolates are summarized in Supplementary Table S3.

### Generic *E. coli* specific qPCR

The 200 bp genomic region located in the *yecE* gene was identified as a generic *E. coli* target using 63 *E. coli* genomes and 137 non-*E. coli* genomes (Supplementary Note S1). However, in 6 out of 45 *Shigella* genomes, *yecE* was found to be present. To test the specificity of the designed primers, *in-silico* amplification showed 99.61% of inclusivity and 90.85% of exclusivity which was lower than expected due to the cross-reactivity with some *Shigella* genomes. The inclusivity results obtained by carrying out *in-vivo* qPCR demonstrated that the *yecE* qPCR assay detects the *yecE* gene across *E. coli* strains (67 strains) with cycle threshold (Ct) values ranging from 11.6 to 23.3 (Supplementary Table S4). No false negatives were observed among the *E. coli* panel confirming the assay’s high inclusivity for *E. coli* detection. The exclusivity panel included a broad spectrum of non-*E. coli* bacterial species, such as *Bacillus cereus*, *Serratia plymuthica*, *Shigella dysenteriae*, *Salmonella enterica*, *Enterococcus faecalis*, and others. None of these 37 non-*E. coli* strains produced a detectable signal with the *yecE* qPCR assay (Supplementary Table S5). This absence of *in vivo* cross-reactivity with non-*E. coli* strains underscores the assay’s specificity for *E. coli*, minimizing the risk of false positives from non-target microorganisms, but not excluding the possibility to pick up closely-related *Shigella* strains which carry the *yecE* gene.

### Colocalization by whole cell ddPCR in wheat flour

Wheat flour samples were inoculated with a single STEC strain, P205 (O103, *stx1*, *eae*, *yecE*), at six different concentration levels. This resulted in a clear gradient of viable cell counts prior to enrichment, with average enumeration values ranging from 3.86 to 1.14 Log_10_ CFU/g for the highest concentrations (Table 2). The enumerations on TBX of the samples inoculated at lower concentrations were below 1.00 Log_10_ CFU/g, therefore their inoculation levels were estimated at 0.26, −0.74, and −1.74 Log_10_ CFU/g. Consistent with the enumeration trends, qPCR targeting *stx1* detected all five replicates as positive at the three highest inoculation levels, whereas detection dropped to three and two positive replicates at intermediate levels and was absent at the lowest inoculum. Similarly, *stx1* Ct values increased from around 19.0 at higher inocula to >23.0 at lower levels, reflecting reduced target gene abundance. Digital droplet PCR colocalization assays (*stx1*/*eae*, *stx1*/O103, and *stx1*/*yecE*) demonstrated colocalization across all replicates at higher inoculation levels but showed a reduction of the number of replicates positive at intermediate concentrations and yielded only negative results at the lowest inoculum. These results indicate a strong correlation of the *stx1* data obtained with qPCR and ddPCR analytical workflows. Additionally, these *stx1* qPCR and ddPCR results obtained with the same set of flour samples inoculated with the P205 strain (Table 2) allowed to estimate the LOD_50_. Despite different sample preparation procedures, the LOD_50_ was found to be at 0.9 CFU/g with lower and upper confidence limits, respectively, at 0.3 and 2.5 CFU/g for both *stx1* qPCR and ddPCR analytical workflows under the tested conditions.

**Table 2:** qPCR and ddPCR results from wheat flour inoculated at different levels with P205 (O103, *stx1*, *eae*, *yecE*).

Number of inoculated flour replicate		5	5	5	5	5	5
Enumeration before enrichment (Log_10_ CFU/g)		3.86	2.29	1.14	0.26	−0.74	−1.74
qPCR	Number of replicates *stx1* positive by qPCR	5	5	5	3	2	0
	qPCR *stx1* Ct	19.2	19.1	19.8	23.0	24.1	ND
ddPCR colocalization	*stx1*/*eae*	5 colocalized	5 colocalized	5 colocalized	3 colocalized	2 colocalized	5 negative
	*stx1*/O103	5 colocalized	5 colocalized	5 colocalized	3 colocalized	2 colocalized	5 negative
	*stx1*/*yecE*	5 colocalized	5 colocalized	5 colocalized	3 colocalized	2 colocalized	5 negative

To further assess the colocalization capabilities of the whole cell ddPCR method, wheat flour samples individually inoculated at two different levels with either P190 (O111, *yecE*) or P202 (O45, *stx1*, *eae*, *yecE*) and analyzed in triplicate by qPCR and ddPCR revealed clear distinctions between the two STEC strains inoculated into flour (Table 3A). Samples inoculated with P190 consistently amplified O111 and *yecE* by qPCR, with average Ct values ranging from 17.3 to 18.7 across both targets and levels of inoculation, while *stx1* remained undetectable, confirming the absence of *stx1* in this strain. In contrast, flour inoculated with P202 produced robust qPCR amplification of O45, *stx1*, *eae,* and *yecE*, with Ct values typically between 16.8 and 19.3, reflecting expected strain-specific target profiles (Table 3A). Quantitative PCR of mixed samples, consisting of combinations of flour samples inoculated with P190 and P202, amplified targets from both strains. However, in the third replicate of the lower inoculation level, O45, *stx1* and *eae* were not detected, suggesting that the P202 strain was below the LOD (Table 3B). Whole cell ddPCR colocalization analysis further supported strain-level discrimination: P190-containing samples showed O111/*yecE* colocalization but no *stx1*-associated colocalization, whereas P202-containing samples demonstrated consistent *stx1*/O45, *stx1*/*yecE*, *stx1*/*eae, eae*/O45 and *eae*/*yecE* colocalization patterns. Mixed samples generated colocalization profiles that reflected contributions from both strains. However, in the first replicate of the lower inoculation level, the *stx1*/*yecE* and *eae*/*yecE* colocalization failed because the QxIDE Manager software was unable to determine the colocalization profile due to an excessive number of *yecE* droplets compared to the *stx1* and *eae* droplets. In the third replicate of the lower inoculation level, all colocalization results for *stx1* were negative, as this target was not detected in any droplets, consistent with the qPCR results. Additionally, O45 and *eae* were not detected in this replicate either, further indicating that the P202 strain fell below the LOD. Collectively, these findings confirm that ddPCR colocalization reliably differentiates STEC strains within flour matrices and accurately detects their presence in mixed-strain scenarios, although limitations may arise when one or more targets are near the LOD, a challenge also encountered by the reference qPCR method.

**Table 3:** qPCR and ddPCR results from wheat flour individually inoculated at different levels with P190 (O111, *yecE*) and P202 (O45, *stx1*, *eae*, *yecE*) (A) and from mixes of the individually inoculated flours at different levels, respectively (B).

(A)					
Flour inoculated with		2.15 Log_10_ CFU/g of P190 (O111, *yecE*)	1.15 Log_10_ CFU/g of P190 (O111, *yecE*)	1.84 Log_10_ CFU/g of P202 (O45, *stx1*, *eae*, *yecE*)	0.84 Log_10_ CFU/g of P202 (O45, *stx1*, *eae*, *yecE*)
qPCR (Ct)	*stx1*	NA^*^	NA	18.9	19.3
	*eae*	NA	NA	17.6	18.1
	O111	18.4	18.7	NA	NA
	O45	NA	NA	16.8	17.2
	*yecE*	17.3	17.9	17.7	18.2
	*stx1*/O45	NA	NA	Colocalized	Colocalized
	*stx1*/*yecE*	NA	NA	Colocalized	Colocalized
	*stx1*/*eae*	NA	NA	Colocalized	Colocalized
	*eae*/O45	NA	NA	Colocalized	Colocalized
	*eae*/*yecE*	NA	NA	Colocalized	Colocalized
	O111/*yecE*	Colocalized	Colocalized	NA	NA

(B)							
Mix 1:1 of flour samples inoculated with		2.15 Log_10_ CFU/g of P190 and 1.84 Log_10_ CFU/g of P202			1.15 Log_10_ CFU/g of P190 and 0.84 Log_10_ CFU/g of P202		
Replicate number		1	2	3	1	2	3
qPCR (Ct)	*stx1*	24.4	20.7	25.7	26.8	20.3	ND
	*eae*	22.8	20.1	23.2	24.3	19.5	ND
	O111	19.5	19.8	17.7	18.2	20.0	19.7
	O45	22.1	19.0	23.1	24.6	18.4	ND
	*yecE*	18.2	17.9	16.6	17.2	18.0	18.5
ddPCR colocalization	*stx1*/O111	Not colocalized	Not colocalized	Not colocalized	Not colocalized	Not colocalized	Negative^a^
	*stx1*/O45	Colocalized	Colocalized	Colocalized	Colocalized	Colocalized	Negative^a^
	*stx1*/*yecE*	Colocalized	Colocalized	Colocalized	Inconclusive	Colocalized	Negative^a^
	*stx1*/*eae*	Colocalized	Colocalized	Colocalized	Colocalized	Colocalized	Negative^b^
	*eae*/O111	Not colocalized	Not colocalized	Not colocalized	Not colocalized	Not colocalized	Negative^c^
	*eae*/O45	Colocalized	Colocalized	Colocalized	Colocalized	Colocalized	Negative^d^
	*eae*/*yecE*	Colocalized	Colocalized	Colocalized	Inconclusive	Colocalized	Negative^e^
	O111/*yecE*	Colocalized	Colocalized	Colocalized	Colocalized	Colocalized	Colocalized

* NA: not applicable.

a Negative as *stx1* not detected.

b Negative as *stx1* and *eae* not detected.

c Negative as *eae* not detected.

d Negative as O45 and *eae* not detected.

e Negative as *eae* not detected.

### Naturally contaminated flours

A total of 19 naturally contaminated flour samples were tested using both qPCR and whole cell ddPCR with a subset of 5 samples undergoing WGS for colony confirmation. Out of these 19 samples, whole cell ddPCR analysis successfully determined in 16 samples whether the *stx* targets were colocalized or not with the other targets (samples 1 to 6, 8, 9, 14, and 16 to 19 for all detected targets; 7, 10, and 12 for all detected targets except *yecE*; Table 4).

**Table 4:** qPCR, ddPCR, and WGS from naturally contaminated flour samples.

Samples	qPCR (average Ct values)							Whole cell ddPCR colocalization												Confirmation by WGS
	*stx1*	*stx2*	*eae*	O103	O145	O45	*yecE*	*stx1*/*yecE*	*stx2*/*yecE*	*stx1*/*eae*	*stx2*/*eae*	*stx1*/O103	*stx2*/O103	*stx1*/O145	*stx1*/O45	O103/*yecE*	O103/*eae*	eae/*yecE*	*eae*/O45	
1	20.9	ND^a^	19.0	ND	20.0	ND	19.5	C^b^	NA	C	NA	NA	NA	C	NA	NA	NA	NA	NA	O145:– *stx1*, *eae*, *yecE*
2	25.7	ND	19.9	ND	25.2	ND	19.9	C	NA	C	NA	NA	NA	C	NA	NA	NA	NA	NA	O145:– *stx1*, *eae*, *yecE*
3	23.1	ND	22.3	ND	22.8	ND	22.4	C	NA	C	NA	NA	NA	C	NA	NA	NA	NA	NA	O145:– *stx1*, *eae*, *yecE*
4	29.0	ND	ND	ND	ND	ND	21.5	C	NA	NA	NA	NA	NA	NA	NA	NA	NA	NA	NA	O105:H20 *stx1*, *yecE*
5	31.1	ND	29.3	ND	29.9	ND	29.0	C	NA	C	NA	NA	NA	C	NA	NA	NA	NA	NA	O145:– *stx1*, *eae*, *yecE*
6	32.2	ND	ND	ND	ND	ND	19.2	NC^c^	NA	NA	NA	NA	NA	NA	NA	NA	NA	NA	NA	Not tested
7	33.5	ND	26.0	ND	ND	25.0	17.1	IC^d^	NA	NC	NA	NA	NA	NA	C	NA	NA	IC	NC	
8	31.2	ND	ND	ND	ND	ND	21.8	C	NA	NA	NA	NA	NA	NA	NA	NA	NA	NA	NA	
9	25.7	ND	ND	26.0	ND	ND	19.8	C	NA	NA	NA	C	NA	NA	NA	C	NA	NA	NA	
10	ND	32.7	ND	28.4	ND	ND	20.3	NA^g^	IC	NA	NA	NA	NC	NA	NA	IC	NA	NA	NA	
11	33.0	ND	ND	ND	ND	ND	20.3	IC	NA	NA	NA	NA	NA	NA	NA	NA	NA	NA	NA	
12	29.0	ND	29.6	32.8	ND	ND	21.8	IC	NA	C	NA	C	NA	NA	NA	C	NA	NC	NA	
13	33.4	ND	ND	ND	ND	ND	19.9	IC	NA	NA	NA	NA	NA	NA	NA	NA	NA	NA	NA	
14	ND	27.3	38.0	ND	ND	ND	21.9	NA	C	NA	N^e^	NA	NA	NA	NA	NA	NA	NA	NA	
15	ND	37.7	28.1	38.7	ND	ND	21.5	NA	N^e^	NA	N^e^	NA	N^e^	NA	NA	NA	N^e^	C	NA	
16	ND	22.1	ND	ND	ND	ND	20.2	NA	C	NA	NA	NA	NA	NA	NA	NA	NA	NA	NA	
17	ND	30.4	26.2	ND	ND	ND	22.7	NA	C	NA	NC	NA	NA	NA	NA	NA	NA	C	NA	
18	32.5	ND	31.9	35.0	ND	ND	27.6	C	NA	N^f^	NA	C	NA	NA	NA	C	N^f^	C	NA	
19	36.1	ND	29.8	32.6	ND	ND	28.1	C	NA	NC	NA	NC	NA	NA	NA	C	C	C	NA	

a ND: not detected.

b C: colocalized.

c NC: not colocalized.

d IC: inconclusive.

e Negative as 1 or 2 targets not detected.

f Negative as number of droplets below the software threshold.

g NA: not applicable.

In samples 1 to 5, analysis of the qPCR, whole cell ddPCR colocalization, and WGS data revealed consistent detection and genetic association patterns across flour samples. In sample 1, low Ct values for O145, *stx1*, *eae*, and *yecE* corresponded with whole cell ddPCR evidence of target colocalization, and WGS confirmed the presence of O145:– strain harboring *stx1*, *eae*, and *yecE*. Same results were observed in three additional samples from the same retail flour batch (samples 2, 3, and 5). Sample 4 yielded positive results for *stx1* and *yecE* positive and exhibited absence of *eae* and Top 7 serogroups by qPCR, which was consistent with its colony WGS classification as O105:H20, carrying *stx1* and *yecE*. The whole cell ddPCR results accurately attributed the colocalization of *stx1* and *yecE*.

For the samples that were not confirmed by WGS, the whole cell ddPCR colocalization analysis enabled the determination of whether the targets detected by qPCR were colocalized or not. For example, in sample 9, O103, *stx1* and *yecE* were detected by qPCR. The whole cell ddPCR analysis showed that these qPCR signals originated from a single strain. In contrast, in sample 19, O103, *stx1*, *eae*, and *yecE* were detected by qPCR and the whole cell ddPCR analysis revealed that these qPCR signals originated from two distinct bacterial strains: one positive for *stx1* and *yecE*, and the other positive for O103, *eae*, and *yecE*.

The use of the *yecE* gene to determine whether the detected STEC targets are located on an *E. coli* genome by whole cell ddPCR was successful for 12 samples (samples 1–5, 8, 9, 14, 16–19; Table 4). Unfortunately, in 5 samples (samples 7, 10–13), the results were inconclusive because the QxIDE Manager software was unable to determine the colocalization profile due to an excessive number of *yecE* droplets compared to the *stx* targets. This was not surprising considering the big difference in qPCR Ct values, as illustrated for sample 7 with Ct values of 33.5 for *stx1* and 17.1 for *yecE*. Finally, limitations driven by the LOD were evident in 3 samples (samples 14, 15, 18) with high qPCR Ct values. In sample 15, *stx2* was detected at a Ct of 37.7 and O103 at a Ct of 38.7, while whole cell ddPCR failed to detect droplets for these targets (Table 4). The same phenomenon was observed in sample 14 with *eae* detected by qPCR at a Ct of 38.0. In sample 18, O103 (Ct 35.0) and *eae* (Ct 31.9) produced insufficient ddPCR droplets to pass the software threshold, again demonstrating that whole cell ddPCR colocalization may be difficult when qPCR Ct values are high. These results demonstrate that successful whole cell ddPCR colocalization critically depends on target concentration, with a heightened risk of failure when one target is notably more prevalent than the others. This risk can be anticipated by examining the qPCR Ct values.

## Discussion

### Whole cell ddPCR in wheat flour

The present work demonstrates the feasibility of applying intact whole cell ddPCR to investigate the co-occurrence of multiple genetic targets within individual STEC bacterial cells in inoculated and naturally contaminated flour samples. One naturally contaminated sample is particularly interesting, as the ddPCR analysis was able to provide more precise information compared to the reference qPCR method, revealing that the sample contained two distinct bacterial strains and that the *stx1* gene was not located in the same strain as the one positive for O103 and *eae* (sample 19). These results demonstrate that the method can effectively reduce the number of presumptive STEC positives in naturally contaminated samples with potentially high levels of background flora. Thus, whole cell ddPCR has the potential to facilitate the decision-making process regarding *stx* positive results in wheat flour and other food matrices, provided that sample preparation is appropriately adapted. The successful colocalization of STEC genes in the presence of high levels of background flora in other food matrices was previously shown to be possible. Both He and coauthors and McMahon and coauthors could accurately detect STEC in the presence of up to 4 Log_10_ higher levels of background flora compared to target cells (He et al., 2020; McMahon et al., 2017).

### Flexibility of the approach

Developing a flexible ddPCR assay where different targets can be analyzed as needed responds to an industry need to accommodate for the different STEC definitions used by different countries. For example in France, samples are considered presumptive STEC positive when *stx1* and/or *stx2*, *eae* and one of the Top 6 serogroups are detected, whereas in Germany, all STEC are considered as potentially pathogenic. This situation hampers harmonized reporting across Europe (Koutsoumanis et al., 2020) and may lead to potential trade issues between neighboring countries. In addition, the ISO/TS-13136:2012 method is currently under revision and the current genetic STEC targets (*stx*, *eae* and the Top 5 serogroups) might change in the future (ISO, 2026). In this context, it is essential to identify genomic regions specific to *E. coli* and to design primers that serve as generic *E. coli* markers in qPCR and ddPCR assays. This approach is necessary to reduce the number of presumptive STEC positives in naturally contaminated samples by accurately interpreting a positive *stx* result which may indicate the presence of an *E. coli* cell carrying the *stx* gene but could also simply arise from a *stx* positive bacteriophage or from free plasmid DNA in the sample. In the present study, we identified the *yecE* gene as a suitable candidate for this purpose. The *yecE* gene was previously reported as a generic *E. coli* gene and corresponds to an integration site of Shiga-toxin 2-encoding bacteriophages, as well as encoding a lipid kinase (Maheux et al., 2011; Tymoszewska and Aleksandrzak-Piekarczyk, 2021). We designed a set of optimal primers and probe, and were able to develop a specific qPCR assay for *E. coli* which allowed us to use the *yecE* gene in all colocalization tests performed throughout the study. However, the *in-silico* evaluation of the primers revealed some cross-reactivity with *Shigella* species which is not surprising, as various authors previously recommended that *Shigella* species should be reclassified as subspecies within *E. coli* (Dif et al., 2025; Cobo-Simón et al., 2023; Parks et al., 2021).

### Potential limitations

Wheat flour inoculated at different levels with one STEC strain (O103, *stx1*, *eae*, *yecE*) and tested in parallel by *stx1* qPCR and ddPCR showed that both analytical workflows have a similar LOD_50_ around 0.9 CFU/g under the tested conditions. LOD is indeed a key factor which poses a challenge for both approaches, as low levels may lead to a negative qPCR result and negative colocalization results. We identified another potential limitation related to the inclusion of a generic *E. coli* marker, such as *yecE*. The background flora, which may contain numerous generic *E. coli*, is enriched alongside the target STEC which may lead to a high number of *yecE* positive droplets compared to a very low count of droplets containing both *yecE* and one of the STEC-specific gene targets. This disparity may cause the ddPCR colocalization software to fail, resulting in an inconclusive report. In the study of Mancusi and coauthors, DNA was used as template for ddPCR and the authors demonstrated that ddPCR can detect STEC very fast, after incubating samples for only 7 h for beef slices and 9 h for sponges (Mancusi et al., 2022). It would be beneficial to investigate shorter incubation times in future whole cell ddPCR experiments on wheat flour to determine if this could facilitate the colocalization of targets with high and low abundances such as *yecE* and *stx*, respectively.

### Integration of whole cell ddPCR into the STEC analytical workflow

The current standard method ISO/TS-13136:2012 is based on a qPCR screening step and a confirmation step by plating which is too cumbersome to be applied in routine monitoring of flour samples, hence it is problematic for the flour milling industry. As discussed by EFSA, there is a pressing need to develop innovative approaches that can provide results in less time, with more sensitivity and specificity than the current reference method (Koutsoumanis et al., 2020). For STEC testing in wheat flour, we propose to keep a first screening by qPCR. If a batch tests positive for *stx* and other genetic targets, as dictated by local regulations, it should undergo more complex screening using whole cell ddPCR, as outlined in the present study. This approach will enhance the differentiation of the genetic backgrounds of STEC strains present in a sample and provide preliminary results to support more accurate food safety risk assessments faster than the confirmation by plating. In the absence of commercially available validated kits for routine use, this approach offers a valuable tool for R&D laboratories to support risk assessments when needed. In this context, additional targets may need to be developed which could be included in a future whole cell ddPCR assay, such as emerging Shiga-toxin subtypes (Wang et al., 2024; Skinner et al., 2016) and other virulence genes which were significantly overrepresented in HUS STEC strains, for example cytolethal distending toxins-encoding genes *cdtA*, *cdtB*, and *cdtC* and the autotransporter serine protease gene *espP* (Bai et al., 2022). Alternatively, the approach could also be used to target other enteropathogenic *E. coli* strains associated with severe disease, but lacking *stx* genes (Söderlund et al., 2023). In conclusion, our results provide a useful data set to support the development of more sophisticated molecular analytical methods to detect and characterize STEC strains which can be applied to wheat flour and other food matrices, provided that sample preparation is appropriately adapted.

## Data Availability

The original contributions presented in the study are publicly available. This data can be found on the NCBI website under the BioProject: PRJNA1475373.

## Footnotes

1
https://pubmlst.org/
