# Supplementary material for: Detection and colocalization of STEC genetic markers in wheat flour using whole cell digital droplet PCR
Source: Front Microbiol. 2026 Jul 24;17:1854595. doi: 10.3389/fmicb.2026.1854595 (PMC13447292; doi:10.3389/fmicb.2026.1854595)
Supplement: Supplementary file 1 [file Data_Sheet_1.docx]

***Frontiers in Microbiology* “Rapid Pathogen Detection in Food Supply Chains"**

Edited by Arun Bhunia, Alexandre Leclercq, Gloria Sánchez Moragas, Dario De Medici.

**Detection and colocalization of STEC genetic markers in wheat flour using whole cell digital droplet PCR**

S. Butot^1^, C. Gaille^1^, L. Michot^1^, M. Seppey^1^, C. Barretto^1^, S. Pruvost^1^, S. Zuber^1^

^1^Institute of Food Safety and Analytical Science, Nestlé Research, Lausanne, Switzerland

**Supplementary material**

**Table S1.** Strains used for the generic and specific *E. coli* gene *in silico* identification**.** This Whole Genome Shotgun project has been deposited at DDBJ/ENA/GenBank under the accession PRJNA1475373.

| Isolate number | Strain number | NCBI BioSample number | Reference culture collection number | Bacterial species |
| --- | --- | --- | --- | --- |
| PIR05730 | E005 | SAMN60637718 | NCTC 13167 | *Escherichia coli* |
| PIR05731 | E191 | SAMN60637719 | ATCC 8739 | *Escherichia coli* |
| PIR05732 | E219 | SAMN60637720 | ATCC 25922 | *Escherichia coli* |
| PIR05733 | E224 | SAMN60637721 | ATCC 11775 | *Escherichia coli* |
| PIR05734 | E467 | SAMN60637722 | ATCC 11229 | *Escherichia coli* |
| PIR05735 | E551 | SAMN60637723 | ATCC 13027 | *Escherichia coli* |
| PIR05736 | E552 | SAMN60637724 | Not applicable | *Shigella sp.*^a^ |
| PIR05737 | E553 | SAMN60637725 | Not applicable | *Shigella sp.* |
| PIR05738 | E554 | SAMN60637726 | Not applicable | *Shigella sp.* |
| PIR05739 | E555 | SAMN60637727 | Not applicable | *Shigella sp.* |
| PIR05740 | E569 | SAMN60637728 | ATCC 12022 | *Shigella flexneri* |
| PIR05742 | E1006 | SAMN60637729 | ATCC 700336 | *Escherichia coli* |
| PIR05743 | E1007 | SAMN60637730 | ATCC 35333 | *Escherichia coli* |
| PIR05744 | E1008 | SAMN60637731 | ATCC 29214 | *Escherichia coli* |
| PIR05745 | E1009 | SAMN60637732 | ATCC 700417 | *Escherichia coli* |
| PIR05746 | E1010 | SAMN60637733 | ATCC 700928 | *Escherichia coli* |
| PIR05747 | E1011 | SAMN60637734 | DSM 10805 | *Escherichia coli* |
| PIR05748 | E1012 | SAMN60637735 | DSM 10798 | *Escherichia coli* |
| PIR05749 | E1013 | SAMN60637736 | DSM 10729 | *Escherichia coli* |
| PIR05750 | E1014 | SAMN60637737 | DSM 10856 | *Escherichia coli* |
| PIR05751 | E1023 | SAMN60637738 | DSM 30083 | *Escherichia coli* |
| PIR05752 | E1032 | SAMN60637739 | ATCC BAA-1427 | *Escherichia coli* |
| PIR05753 | E1079 | SAMN60637740 | ATCC 23631 | *Escherichia coli* |
| PIR05754 | P025 | SAMN60637741 | Not applicable | *Escherichia coli* |
| PIR05755 | P040 | SAMN60637742 | Not applicable | *Escherichia coli*^b^ |
| PIR05756 | P042 | SAMN60637743 | Not applicable | *Escherichia coli*^b^ |
| PIR05757 | P043 | SAMN60637744 | Not applicable | *Escherichia coli*^b^ |
| PIR05758 | P046 | SAMN60637745 | Not applicable | *Escherichia coli* ^c^ |
| PIR05759 | P047 | SAMN60637746 | Not applicable | *Escherichia coli*^b^ |
| PIR05760 | P049 | SAMN60637747 | Not applicable | *Escherichia coli*^b^ |
| PIR05761 | P050 | SAMN60637748 | Not applicable | *Escherichia coli* ^a^ |
| PIR05762 | P052 | SAMN60637749 | Not applicable | *Escherichia coli*^b^ |
| PIR05763 | P058 | SAMN60637750 | Not applicable | *Escherichia coli* ^a^ |
| PIR05764 | P060 | SAMN60637751 | Not applicable | *Escherichia coli* ^a^ |
| PIR05765 | P077 | SAMN60637752 | Not applicable | *Escherichia coli* ^a^ |
| PIR05766 | P078 | SAMN60637753 | Not applicable | *Escherichia coli* ^a^ |
| PIR05767 | P080 | SAMN60637754 | Not applicable | *Escherichia coli*^b^ |
| PIR05768 | P085 | SAMN60637755 | Not applicable | *Escherichia coli* |
| PIR05769 | P088 | SAMN60637756 | Not applicable | *Escherichia coli*^b^ |
| PIR05770 | P106 | SAMN60637757 | Not applicable | *Escherichia coli*^b^ |
| PIR05771 | P125 | SAMN60637758 | Not applicable | *Escherichia coli* |
| PIR05772 | P126 | SAMN60637759 | Not applicable | *Escherichia coli* |
| PIR05773 | P138 | SAMN60637760 | DSM 17076 | *Escherichia coli* |
| PIR05774 | P139 | SAMN60637761 | ATCC 700728 | *Escherichia coli* |
| PIR05775 | P141 | SAMN60637762 | Not applicable | *Escherichia coli* |
| PIR05776 | P149 | SAMN60637763 | Not applicable | *Escherichia coli* ^a^ |
| PIR05777 | P150 | SAMN60637764 | Not applicable | *Escherichia coli*^b^ |
| PIR05778 | P151 | SAMN60637765 | Not applicable | *Escherichia coli*^b^ |
| PIR05779 | P152 | SAMN60637766 | Not applicable | *Escherichia coli* ^c^ |
| PIR05780 | P153 | SAMN60637767 | Not applicable | *Escherichia coli* ^a^ |
| PIR05781 | P154 | SAMN60637768 | Not applicable | *Escherichia coli* ^a^ |
| PIR05782 | P155 | SAMN60637769 | Not applicable | *Escherichia coli* |
| PIR05783 | P190 | SAMN60637770 | ATCC 29552 | *Escherichia coli* |
| PIR05784 | P191 | SAMN60637771 | ATCC BAA-2190 | *Escherichia coli* |
| PIR05785 | P192 | SAMN60637772 | ATCC BAA-2212 | *Escherichia coli* |
| PIR05787 | P194 | SAMN60637773 | ATCC BAA-2216 | *Escherichia coli* |
| PIR05788 | P195 | SAMN60637774 | ATCC BAA-2649 | *Escherichia coli* |
| PIR05789 | P199 | SAMN60637775 | ATCC 43890 | *Escherichia coli* ^a^ |
| PIR05790 | P200 | SAMN60637776 | ATCC BAA-2326 | *Escherichia coli* ^b^ |
| PIR05791 | P201 | SAMN60637777 | CDC 99-3311 | *Escherichia coli* ^c^ |
| PIR05792 | P202 | SAMN60637778 | CDC 00-3039 | *Escherichia coli* ^a^ |
| PIR05793 | P203 | SAMN60637779 | CDC 02-3211 | *Escherichia coli* ^b^ |
| PIR05794 | P204 | SAMN60637780 | CDC 03-3014 | *Escherichia coli* ^c^ |
| PIR05795 | P205 | SAMN60637781 | CDC 06-3008 | *Escherichia coli* ^a^ |
| PIR05796 | P206 | SAMN60637782 | CDC 2010c-3114 | *Escherichia coli* ^c^ |
| PIR05797 | P207 | SAMN60637783 | ATCC 35150 | *Escherichia coli* ^c^ |
| PIR05798 | E1015 | SAMN60637784 | DSM 10744 | *Escherichia coli* |
| PIR05799 | E223 | SAMN60637785 | ATCC 23716 | *Escherichia coli* |

^a^Strain *stx1* positive, ^b^Strain *stx2* positive, ^c^Strain *stx1* and *stx2* positive.

**Table S2.** Public genome identifiers that were used as the negative group in the BLAST search to highlight *E. coli*-specific regions *in silico*.

| GenBank Accession | Taxon |
| --- | --- |
| GCA_002927275.1 | *Arthrobacter pityocampae* |
| GCA_008087155.1 | *Bacillus anthracis* |
| GCA_016650515.1 | *Bacillus sp. TH57* |
| GCA_016650525.1 | *Bacillus sp. TH59* |
| GCA_016650495.1 | *Bacillus sp. TH86* |
| GCA_009019275.1 | *Bacteroides uniformis* |
| GCA_009019315.1 | *Bacteroides uniformis* |
| GCA_009019735.1 | *Bacteroides uniformis* |
| GCA_000525915.1 | *Brochothrix campestris* |
| GCA_001715655.1 | *Brochothrix thermosphacta* |
| GCA_900332065.1 | *Brochothrix thermosphacta* |
| GCA_001703535.1 | *Brochothrix thermosphacta* |
| GCA_001715665.1 | *Brochothrix thermosphacta* |
| GCA_001716015.1 | *Brochothrix thermosphacta* |
| GCA_002381265.1 | *Brochothrix thermosphacta* |
| GCA_002442515.1 | *Brochothrix thermosphacta* |
| GCA_009295555.1 | *Brochothrix thermosphacta* |
| GCA_002931295.1 | *Clavibacter michiganensis* |
| GCA_001876145.1 | *Corynebacterium diphtheriae* |
| GCA_001876165.1 | *Corynebacterium diphtheriae* |
| GCA_001876245.1 | *Corynebacterium diphtheriae* |
| GCA_900475605.1 | *Corynebacterium ulcerans* |
| GCA_003097655.1 | *Flavobacterium laiguense* |
| GCA_007097385.1 | *Flavobacterium sp.* |
| GCA_003265355.1 | *Glycomyces dulcitolivorans* |
| GCA_000822385.1 | *Klebsiella pneumoniae* |
| GCA_001182385.1 | *Klebsiella pneumoniae* |
| GCA_002262385.1 | *Klebsiella pneumoniae* |
| GCA_002386225.2 | *Klebsiella pneumoniae* |
| GCA_002812385.1 | *Klebsiella pneumoniae* |
| GCA_004832385.1 | *Klebsiella pneumoniae* |
| GCA_011742385.1 | *Klebsiella pneumoniae* |
| GCA_012952385.1 | *Klebsiella pneumoniae* |
| GCA_013423825.1 | *Klebsiella pneumoniae* |
| GCA_015238555.1 | *Klebsiella pneumoniae* |
| GCA_015238655.1 | *Klebsiella pneumoniae* |
| GCA_015238895.1 | *Klebsiella pneumoniae* |
| GCA_015992385.1 | *Klebsiella pneumoniae* |
| GCA_017922385.1 | *Klebsiella pneumoniae* |
| GCA_004312385.1 | *Klebsiella variicola* |
| GCA_008831305.1 | *Kocuria sp.* |
| GCA_017571325.1 | *Listeria monocytogenes* |
| GCA_017571345.1 | *Listeria monocytogenes* |
| GCA_017571385.1 | *Listeria monocytogenes* |
| GCA_017571395.1 | *Listeria monocytogenes* |
| GCA_017571425.1 | *Listeria monocytogenes* |
| GCA_017571445.1 | *Listeria monocytogenes* |
| GCA_900123685.1 | *Mycobacterium tuberculosis* |
| GCA_002802815.1 | *Mycobacteroides abscessus* |
| GCA_008638365.1 | *Nocardiopsis quinghaiensis* |
| GCA_002931575.1 | *Pseudoclavibacter sp.* |
| GCA_002931555.1 | *Pseudoclavibacter sp.* |
| GCA_002931455.1 | *Pseudoclavibacter sp.* |
| GCA_002931395.1 | *Pseudoclavibacter sp.* |
| GCA_000760755.1 | *Rhodococcus fascians* |
| GCA_024124965.1 | *Riemerella anatipestifer* |
| GCA_001652385.2 | *Salmonella enterica* |
| GCA_008923865.1 | *Salmonella enterica* |
| GCA_000238435.2 | *Salmonella enterica* |
| GCA_000238455.3 | *Salmonella enterica* |
| GCA_000238495.2 | *Salmonella enterica* |
| GCA_000238515.2 | *Salmonella enterica* |
| GCA_000238535.2 | *Salmonella enterica* |
| GCA_000238555.2 | *Salmonella enterica* |
| GCA_000623815.2 | *Salmonella enterica* |
| GCA_000623835.2 | *Salmonella enterica* |
| GCA_000623855.2 | *Salmonella enterica* |
| GCA_000623875.2 | *Salmonella enterica* |
| GCA_000623895.2 | *Salmonella enterica* |
| GCA_001082385.1 | *Salmonella enterica* |
| GCA_001092385.1 | *Salmonella enterica* |
| GCA_001172385.1 | *Salmonella enterica* |
| GCA_001238265.1 | *Salmonella enterica* |
| GCA_001238285.1 | *Salmonella enterica* |
| GCA_001238365.1 | *Salmonella enterica* |
| GCA_001238375.1 | *Salmonella enterica* |
| GCA_001623805.1 | *Salmonella enterica* |
| GCA_001623825.2 | *Salmonella enterica* |
| GCA_001623845.1 | *Salmonella enterica* |
| GCA_001832385.1 | *Salmonella enterica* |
| GCA_002238275.1 | *Salmonella enterica* |
| GCA_002386165.1 | *Salmonella enterica* |
| GCA_002386325.1 | *Salmonella enterica* |
| GCA_004323815.1 | *Salmonella enterica* |
| GCA_004323855.1 | *Salmonella enterica* |
| GCA_012223825.1 | *Salmonella enterica* |
| GCA_016923815.1 | *Salmonella enterica* |
| GCA_016923835.1 | *Salmonella enterica* |
| GCA_016923845.1 | *Salmonella enterica* |
| GCA_016923875.1 | *Salmonella enterica* |
| GCA_016923895.1 | *Salmonella enterica* |
| GCA_001061275.1 | *Shigella boydii* |
| GCA_002247435.1 | *Shigella boydii* |
| GCA_002248875.1 | *Shigella boydii* |
| GCA_002735445.1 | *Shigella boydii* |
| GCA_002949455.1 | *Shigella boydii* |
| GCA_002949495.1 | *Shigella boydii* |
| GCA_002968245.1 | *Shigella boydii* |
| GCA_003572225.1 | *Shigella boydii* |
| GCA_003572455.1 | *Shigella boydii* |
| GCA_013394535.1 | *Shigella boydii* |
| GCA_000211935.2 | *Shigella dysenteriae* |
| GCA_001932995.2 | *Shigella dysenteriae* |
| GCA_002949415.1 | *Shigella dysenteriae* |
| GCA_002949755.1 | *Shigella dysenteriae* |
| GCA_002949915.1 | *Shigella dysenteriae* |
| GCA_002950055.1 | *Shigella dysenteriae* |
| GCA_002950175.1 | *Shigella dysenteriae* |
| GCA_002968215.1 | *Shigella dysenteriae* |
| GCA_003336975.1 | *Shigella dysenteriae* |
| GCA_022354145.1 | *Shigella dysenteriae* |
| GCA_902502535.1 | *Shigella flexneri* |
| GCA_902503995.1 | *Shigella flexneri* |
| GCA_000815805.1 | *Shigella flexneri* |
| GCA_000566465.1 | *Shigella flexneri* |
| GCA_003572695.1 | *Shigella flexneri* |
| GCA_003572845.1 | *Shigella flexneri* |
| GCA_009743235.1 | *Shigella flexneri* |
| GCA_009743685.1 | *Shigella flexneri* |
| GCA_900486105.1 | *Shigella flexneri* |
| GCA_900486305.1 | *Shigella flexneri* |
| GCA_001250715.1 | *Shigella sonnei* |
| GCA_001257915.1 | *Shigella sonnei* |
| GCA_001258315.1 | *Shigella sonnei* |
| GCA_900157525.1 | *Shigella sonnei* |
| GCA_900157745.1 | *Shigella sonnei* |
| GCA_900158055.1 | *Shigella sonnei* |
| GCA_900158605.1 | *Shigella sonnei* |
| GCA_900159815.1 | *Shigella sonnei* |
| GCA_900484965.1 | *Shigella sonnei* |
| GCA_900485095.1 | *Shigella sonnei* |
| GCA_012926745.1 | *Staphylococcus capitis* |
| GCA_001705245.1 | *Staphylococcus haemolyticus* |
| GCA_023534745.1 | *Streptomyces sp.* |
| GCA_023184515.1 | *Streptomyces sp.* |
| GCA_014748315.1 | *Streptomyces sp.* |
| GCA_018638305.1 | *Streptomyces sp.* |

**Table S3.** Assembly quality metrics of the sequenced isolates.

| Sample Name | % BUSCO score (S,D,F,M)* | n contigs | Assembly size in bp | % GC content | Genome coverage |
| --- | --- | --- | --- | --- | --- |
| PIR05730 | 99.8, 0.2, 0.0, 0.0 | 128 | 4693959 | 50 | *153* |
| PIR05731 | 99.5, 0.5, 0.0, 0.0 | 90 | 4647957 | 50 | *187* |
| PIR05732 | 99.5, 0.5, 0.0, 0.0 | 89 | 5121506 | 50 | *182* |
| PIR05733 | 99.5, 0.5, 0.0, 0.0 | 52 | 4979223 | 50 | *177* |
| PIR05734 | 99.5, 0.5, 0.0, 0.0 | 73 | 4837163 | 50 | *199* |
| PIR05735 | 99.5, 0.5, 0.0, 0.0 | 225 | 5234096 | 50 | *210* |
| PIR05736 | 98.6, 0.2, 0.0, 1.2 | 520 | 3990730 | 50 | *214* |
| PIR05737 | 99.5, 0.2, 0.2, 0.1 | 443 | 4282672 | 50 | *205* |
| PIR05738 | 99.5, 0.2, 0.2, 0.1 | 435 | 4360018 | 50 | *102* |
| PIR05739 | 99.5, 0.2, 0.2, 0.1 | 419 | 4336650 | 50 | *114* |
| PIR05740 | 99.1, 0.5, 0.0, 0.4 | 286 | 4458582 | 50 | *108* |
| PIR05742 | 99.5, 0.5, 0.0, 0.0 | 60 | 5189530 | 50 | *117* |
| PIR05743 | 99.5, 0.5, 0.0, 0.0 | 88 | 4931480 | 50 | *113* |
| PIR05744 | 99.5, 0.5, 0.0, 0.0 | 142 | 5153876 | 50 | *122* |
| PIR05745 | 99.5, 0.5, 0.0, 0.0 | 76 | 5225194 | 50 | *145* |
| PIR05746 | 99.5, 0.5, 0.0, 0.0 | 94 | 5135406 | 50 | *144* |
| PIR05747 | 99.5, 0.5, 0.0, 0.0 | 67 | 5205537 | 50 | *147* |
| PIR05748 | 99.5, 0.5, 0.0, 0.0 | 124 | 5400985 | 50 | *103* |
| PIR05749 | 99.5, 0.5, 0.0, 0.0 | 80 | 5065445 | 50 | *136* |
| PIR05750 | 99.5, 0.5, 0.0, 0.0 | 64 | 5181008 | 50 | *134* |
| PIR05751 | 99.5, 0.5, 0.0, 0.0 | 46 | 4980489 | 50 | *157* |
| PIR05752 | 99.5, 0.5, 0.0, 0.0 | 68 | 4823723 | 50 | *176* |
| PIR05753 | 99.5, 0.5, 0.0, 0.0 | 82 | 4630291 | 50 | *230* |
| PIR05754 | 99.5, 0.5, 0.0, 0.0 | 123 | 5366893 | 50 | *133* |
| PIR05755 | 99.5, 0.5, 0.0, 0.0 | 114 | 5120224 | 50 | *133* |
| PIR05756 | 99.3, 0.7, 0.0, 0.0 | 122 | 5059731 | 50 | *134* |
| PIR05757 | 99.5, 0.5, 0.0, 0.0 | 168 | 5559200 | 50 | *144* |
| PIR05758 | 99.5, 0.5, 0.0, 0.0 | 145 | 5163510 | 50 | *125* |
| PIR05759 | 99.5, 0.5, 0.0, 0.0 | 175 | 5536969 | 50 | *131* |
| PIR05760 | 99.5, 0.5, 0.0, 0.0 | 117 | 5201350 | 50 | *127* |
| PIR05761 | 99.5, 0.5, 0.0, 0.0 | 221 | 5611037 | 50 | *129* |
| PIR05762 | 99.3, 0.7, 0.0, 0.0 | 195 | 5268745 | 50 | *129* |
| PIR05763 | 99.5, 0.5, 0.0, 0.0 | 80 | 5326425 | 50 | *160* |
| PIR05764 | 99.5, 0.5, 0.0, 0.0 | 45 | 5167244 | 50 | *150* |
| PIR05765 | 99.5, 0.5, 0.0, 0.0 | 42 | 5168005 | 50 | *167* |
| PIR05766 | 99.5, 0.5, 0.0, 0.0 | 109 | 5306630 | 50 | *138* |
| PIR05767 | 99.5, 0.5, 0.0, 0.0 | 132 | 5427885 | 50 | *147* |
| PIR05768 | 99.5, 0.5, 0.0, 0.0 | 67 | 4783688 | 50 | *156* |
| PIR05769 | 99.3, 0.7, 0.0, 0.0 | 124 | 5047883 | 50 | *185* |
| PIR05770 | 99.5, 0.5, 0.0, 0.0 | 71 | 5155480 | 50 | *112* |
| PIR05771 | 99.5, 0.5, 0.0, 0.0 | 85 | 4925775 | 50 | *120* |
| PIR05772 | 99.5, 0.5, 0.0, 0.0 | 79 | 4865380 | 50 | *116* |
| PIR05773 | 99.5, 0.5, 0.0, 0.0 | 128 | 5264095 | 50 | *134* |
| PIR05774 | 99.5, 0.5, 0.0, 0.0 | 140 | 5300360 | 50 | *121* |
| PIR05775 | 99.5, 0.5, 0.0, 0.0 | 162 | 5320530 | 50 | *132* |
| PIR05776 | 99.3, 0.5, 0.2, 0.0 | 156 | 5344043 | 50 | *147* |
| PIR05777 | 99.5, 0.5, 0.0, 0.0 | 110 | 5062077 | 50 | *168* |
| PIR05778 | 99.5, 0.5, 0.0, 0.0 | 101 | 5070156 | 50 | *98* |
| PIR05779 | 99.5, 0.5, 0.0, 0.0 | 120 | 5366173 | 50 | *107* |
| PIR05780 | 99.5, 0.5, 0.0, 0.0 | 161 | 5306707 | 50 | *105* |
| PIR05781 | 99.3, 0.7, 0.0, 0.0 | 205 | 5613081 | 50 | *120* |
| PIR05782 | 99.5, 0.5, 0.0, 0.0 | 61 | 4937383 | 50 | *91* |
| PIR05783 | 99.5, 0.5, 0.0, 0.0 | 69 | 5018741 | 50 | *107* |
| PIR05784 | 99.5, 0.5, 0.0, 0.0 | 246 | 4922106 | 50 | *127* |
| PIR05785 | 99.5, 0.5, 0.0, 0.0 | 37 | 4964702 | 50 | *116* |
| PIR05787 | 99.5, 0.5, 0.0, 0.0 | 34 | 4672765 | 50 | *105* |
| PIR05788 | 99.5, 0.5, 0.0, 0.0 | 101 | 5221811 | 50 | *108* |
| PIR05789 | 99.5, 0.5, 0.0, 0.0 | 131 | 5255672 | 50 | *140* |
| PIR05790 | 99.5, 0.5, 0.0, 0.0 | 112 | 5313673 | 50 | *122* |
| PIR05791 | 99.5, 0.5, 0.0, 0.0 | 146 | 5273704 | 50 | *126* |
| PIR05792 | 99.5, 0.5, 0.0, 0.0 | 167 | 5295775 | 50 | *134* |
| PIR05793 | 99.5, 0.5, 0.0, 0.0 | 169 | 5181506 | 50 | *151* |
| PIR05794 | 99.5, 0.5, 0.0, 0.0 | 211 | 5566612 | 50 | *91* |
| PIR05795 | 99.3, 0.7, 0.0, 0.0 | 184 | 5442846 | 50 | *85* |
| PIR05796 | 99.8, 0.2, 0.0, 0.0 | 163 | 5349930 | 50 | *97* |
| PIR05797 | 99.5, 0.5, 0.0, 0.0 | 122 | 5347286 | 50 | *96* |
| PIR05798 | 99.5, 0.5, 0.0, 0.0 | 52 | 5274351 | 50 | *98* |
| PIR05799 | 99.5, 0.5, 0.0, 0.0 | 69 | 4701639 | 50 | *109* |

**Table S4.** Strains used for the *in vivo* inclusivity assessment of the *yecE* qPCR assay.

| Strain number | Bacterial species | Source | *yecE* results (Ct) |
| --- | --- | --- | --- |
| E005 | *Escherichia coli* | NCTC 13167 | 15.66 |
| E191 | *Escherichia coli* | ATCC 8739 | 11.98 |
| E219 | *Escherichia coli* | ATCC 25922 | 15.64 |
| E224 | *Escherichia coli* | ATCC 11775 | 15.53 |
| E467 | *Escherichia coli* | ATCC 11229 | 12.58 |
| E551 | *Escherichia coli* | ATCC 13027 | 13.96 |
| E1006 | *Escherichia coli* | ATCC 700336 | 15.56 |
| E1007 | *Escherichia coli* | ATCC 35333 | 11.79 |
| E1008 | *Escherichia coli* | ATCC 29214 | 12.07 |
| E1009 | *Escherichia coli* | ATCC 700417 | 14.95 |
| E1010 | *Escherichia coli* | ATCC 700928 | 14.86 |
| E1011 | *Escherichia coli* | DSM 10805 | 14.99 |
| E1012 | *Escherichia coli* | DSM 10798 | 15.45 |
| E1013 | *Escherichia coli* | DSM 10729 | 15.03 |
| E1014 | *Escherichia coli* | DSM 10856 | 15.06 |
| E1015 | *Escherichia coli* | DSM 10744 | 14.95 |
| E1023 | *Escherichia coli* | DSM 30083 | 15.52 |
| E1032 | *Escherichia coli* | BAA-1427 | 11.56 |
| E1079 | *Escherichia coli* | ATCC 23631 | 12.25 |
| P025 | *Escherichia coli* | Unknown | 12.86 |
| P026 | *Escherichia coli* ^c^ | CDC | 13.09 |
| P037 | *Escherichia coli* ^c^ | Unknown | 13.12 |
| P040 | *Escherichia coli* ^b^ | University of Zurich | 13.08 |
| P042 | *Escherichia coli* ^b^ | University of Zurich | 13.04 |
| P043 | *Escherichia coli* ^b^ | University of Zurich | 13.04 |
| P046 | *Escherichia coli* ^c^ | University of Zurich | 16.77 |
| P047 | *Escherichia coli* ^b^ | University of Zurich | 13.20 |
| P049 | *Escherichia coli* ^b^ | University of Zurich | 14.14 |
| P050 | *Escherichia coli* ^a^ | University of Zurich | 12.98 |
| P052 | *Escherichia coli* ^b^ | University of Zurich | 13.29 |
| P058 | *Escherichia coli* ^a^ | University of Zurich | 14.28 |
| P060 | *Escherichia coli* ^a^ | University of Zurich | 13.39 |
| P077 | *Escherichia coli* ^a^ | Unknown | 13.07 |
| P078 | *Escherichia coli* ^a^ | Unknown | 13.13 |
| P080 | *Escherichia coli* ^b^ | University of Zurich | 13.07 |
| P083 | *Escherichia coli* ^a^ | University of Zurich | 12.99 |
| P085 | *Escherichia coli* | University of Zurich | 23.27 |
| P088 | *Escherichia coli* ^b^ | University of Zurich | 13.24 |
| P106 | *Escherichia coli* ^b^ | University of Zurich | 13.00 |
| P125 | *Escherichia coli* | UMD864 | 17.80 |
| P126 | *Escherichia coli* | UMD870 | 17.30 |
| P138 | *Escherichia coli* | DSM 17076 | 13.14 |
| P139 | *Escherichia coli* | ATCC 700728 | 13.62 |
| P141 | *Escherichia coli* | IFSH | 13.34 |
| P149 | *Escherichia coli* ^a^ | IFSH | 12.10 |
| P150 | *Escherichia coli* ^b^ | IFSH | 12.41 |
| P151 | *Escherichia coli* ^b^ | IFSH | 12.23 |
| P152 | *Escherichia coli* ^c^ | IFSH | 12.81 |
| P153 | *Escherichia coli* ^a^ | IFSH | 12.27 |
| P154 | *Escherichia coli* ^a^ | IFSH | 12.17 |
| P155 | *Escherichia coli* | IFSH | 12.40 |
| P190 | *Escherichia coli* | ATCC 29552 | 11.76 |
| P191 | *Escherichia coli* | ATCC BAA-2190 | 12.70 |
| P192 | *Escherichia coli* | ATCC BAA-2212 | 15.32 |
| P194 | *Escherichia coli* | ATCC BAA-2216 | 15.77 |
| P195 | *Escherichia coli* | ATCC BAA-2649 | 12.19 |
| P199 | *Escherichia coli* ^a^ | ATCC 43890 | 13.24 |
| P200 | *Escherichia coli* ^b^ | ATCC BAA-2326 | 12.03 |
| P201 | *Escherichia coli* ^c^ | CDC 99-3311 | 13.07 |
| P202 | *Escherichia coli* ^a^ | CDC 00-3039 | 12.50 |
| P203 | *Escherichia coli* ^b^ | CDC 02-3211 | 12.97 |
| P204 | *Escherichia coli* ^c^ | CDC 03-3014 | 13.14 |
| P205 | *Escherichia coli* ^a^ | CDC 06-3008 | 12.43 |
| P206 | *Escherichia coli* ^c^ | CDC 2010c-3114 | 13.09 |
| P207 | *Escherichia coli* ^c^ | ATCC 35150 | 12.59 |
| P212 | *Escherichia coli* ^b^ | University of Zurich | 12.63 |
| P213 | *Escherichia coli* ^b^ | University of Zurich | 12.97 |

^a^ strain *stx1* positive, ^b^ strain *stx2* positive, ^c^ strain *stx1* and *stx2* positive.

**Table S5.** Strains used for the *in vivo* exclusivity assessment of the *yecE* qPCR assay.

| Strain number | Bacterial species | Source | *yecE* results (Ct) |
| --- | --- | --- | --- |
| B412 | *Bacillus cereus* | ATCC 33019 | Undetected |
| B413 | *Bacillus subtilis* | ATCC 6051 | Undetected |
| E213 | *Serratia plymuthica* | DSM 4540 | Undetected |
| E214 | *Serratia plymuthica* | DSM 30127 | Undetected |
| E500 | *Enterobacter sakazakii* | Unknown | Undetected |
| E552 | *Shigella dysenteriae*^a^ | Unknown | Undetected |
| E553 | *Shigella dysenteriae* | Unknown | Undetected |
| E554 | *Shigella dysenteriae* | Unknown | Undetected |
| E555 | *Shigella dysenteriae* | Unknown | Undetected |
| E569 | *Shigella flexneri* | ATCC 12022 | Undetected |
| E585 | *Klebsiella oxytoca* | ATCC 13182 | Undetected |
| E586 | *Serratia marescens* | ATCC 13880 | Undetected |
| E587 | *Edwardsiella tarda* | ATCC 15469 | Undetected |
| E599 | *Citrobacter freundii* | ATCC 8090 | Undetected |
| E1022 | *Enterobacter cloaceae* | DSM 30054 | Undetected |
| E1024 | *Hafnia alvei* | DSM 30163 | Undetected |
| E1025 | *Klebsiella pneumoniae* | DSM 30104 | Undetected |
| E1044 | *Citrobacter freundii* | DSM 30039 | Undetected |
| E1045 | *Cronobacter sakazakii* | DSM 4485 | Undetected |
| E1046 | *Edwardsiella tarda* | DSM 30052 | Undetected |
| E1075 | *Cronobacter sakazakii* | ATCC 29544 | Undetected |
| E1077 | *Enterobacter cloacae* | ATCC 13047 | Undetected |
| L412 | *Listeria innocua* | DSM 20649 | Undetected |
| L505 | *Listeria monocytogenes* | DSM 20600 | Undetected |
| S240 | *Salmonella enterica* serovar Tess | Unknown | Undetected |
| S291 | *Salmonella enterica* serovar Adelaide | Unknown | Undetected |
| S649 | *Salmonella enterica* serovar Tennessee | ATCC 10722 | Undetected |
| S650 | *Salmonella enterica* serovar Anatum | ATCC BAA-1592 | Undetected |
| S651 | *Salmonella enterica* serovar Montevideo | ATCC BAA-710 | Undetected |
| S652 | *Salmonella enterica* serovar Agona | ATCC 51957 | Undetected |
| S654 | *Salmonella enterica* serovar Enteritidis | ATCC BAA-708 | Undetected |
| STR045 | *Enterococcus faecalis* | ATCC 2320 | Undetected |
| STR046 | *Enterococcus faecium* | ATCC 2367 | Undetected |
| STR047 | *Enterococcus faecium* | ATCC 2380 | Undetected |
| STR048 | *Enterococcus faecium* | ATCC 8043 | Undetected |
| STR076 | *Enterococcus faecalis* | ATCC 29212 | Undetected |
| STR077 | *Enterococcus faecalis* | DSM 20478 | Undetected |

^a^ strain *stx1* positive.

**Note S1.** The 200-bp region used to design the *yecE* primers and probe.

CACAGATGACTTCCGCTTCTGTTTTAAGTTTCCGGC

GACCATTTCGCATCAGGCAGCATTACGACATTGCGA

TGATTTAGTGACTGAATTTTTGACCCGCATGTCACC

GTTGGCTCCGCGCATTGGGCAATACTGGCTGCAACT

GCCTGCCACATTCGGCCCACGGGAGCTGCCTGCGCT

TTGGCATTTTCTCGATTCTC

Binding region of primers and probes (green: fwd primer; blue: probe; pink: rev primer).
